# Supplementary material for: End Sequence Analysis Toolkit (ESAT) expands the extractable information from single-cell RNA-seq data
Source: Genome Res. 2016 Oct;26(10):1397–410. doi: 10.1101/gr.207902.116 (PMC5052061; doi:10.1101/gr.207902.116)
Supplement: Supplemental Material [file supp_gr.207902.116_Supp_Materials.tar.gz › ESAT-esat_v0.1/doc/html/classbroad_1_1core_1_1overlaputils_1_1_gene_set_intersect.html]

ESAT: broad.core.overlaputils.GeneSetIntersect Class Reference


ESAT


- Main Page
- Related Pages
- Packages
- Classes
- Files

- Class List
- Class Index
- Class Hierarchy
- Class Members

All Classes Namespaces Files Functions Variables Enumerator Pages

- broad
- core
- overlaputils
- GeneSetIntersect

Static Public Member Functions |
List of all members

broad.core.overlaputils.GeneSetIntersect Class Reference

|  |  |
| --- | --- |
| Static Public Member Functions | |
| static Map< String, Map< Gene,   Collection< Gene > > > | mapGenesToOverlappers (Map< String, Collection< Gene >> genes, Map< String, Collection< Gene >> intersectSet) |
|  | |
| static Map< String, Collection  < Gene > > | getOverlap (Map< String, Collection< Gene >> genes, Map< String, Collection< Gene >> intersectSet) |
|  | |
| static Map< String, Collection  < Gene > > | getOverlap (Map< String, Collection< Gene >> genes, Map< String, Collection< Gene >> intersectSet, boolean verbose) |
|  | |
| static Map< String, Collection  < Gene > > | getOverlap (Map< String, Collection< Gene >> genes, Map< String, Collection< Gene >> intersectSet, Map< String, Collection< Gene >> excludeSet) |
|  | |
| static Map< String, Collection  < Gene > > | getOverlap (Map< String, Collection< Gene >> genes, Map< String, Collection< Gene >> intersectSet, Map< String, Collection< Gene >> excludeSet, boolean verbose) |
|  | |
| static int | countOverlappers (Map< String, Collection< Gene >> regionsToCount, Map< String, Collection< Gene >> regionsToOverlapWith) |
|  | |
| static int | countOverlappers (Map< String, Collection< Gene >> regionsToCount, Map< String, Collection< Gene >> regionsToOverlapWith, Map< String, Collection< Gene >> excludeSet) |
|  | |
| static Map< String, Collection  < Gene > > | getOverlapWithRandomizedPositions (Map< String, Collection< Gene >> regions, Map< String, Collection< Gene >> intersectGenes) |
|  | |
| static Map< String, Collection  < Gene > > | getOverlapWithRandomizedPositions (Map< String, Collection< Gene >> regions, Map< String, Collection< Gene >> intersectGenes, Map< String, Collection< Gene >> excludeFeatures) |
|  | |
| static int | numOverlappingBases (Map< String, Collection< Gene >> genes, Map< String, Collection< Gene >> intersectSet) |
|  | |
| static int | numOverlappingBases (Map< String, Collection< Gene >> genes, Map< String, Collection< Gene >> intersectSet, Map< String, Collection< Gene >> excludeSet) |
|  | |
| static int | numOverlappingGenes (Map< String, Collection< Gene >> genes, Map< String, Collection< Gene >> intersectSet) |
|  | |
| static int | numOverlappingGenes (Map< String, Collection< Gene >> genes, Map< String, Collection< Gene >> intersectSet, Map< String, Collection< Gene >> excludeGenes) |
|  | |
| static void | writeOverlap (String geneFile, String intersectFile, String outFile, boolean randomizePositionsWithinEachGene) throws IOException |
|  | |
| static void | writeOverlap (String geneFile, String intersectFile, String excludeFile, String outFile, boolean randomizePositionsWithinEachGene) throws IOException |
|  | |
| static void | writeOverlap (Map< String, Collection< Gene >> genes, Map< String, Collection< Gene >> intersectSet, String outFile, boolean randomizePositionsWithinEachGene) throws IOException |
|  | |
| static void | writeOverlap (Map< String, Collection< Gene >> genes, Map< String, Collection< Gene >> intersectSet, Map< String, Collection< Gene >> excludeSet, String outFile, boolean randomizePositionsWithinEachGene) throws IOException |
|  | |
| static void | writeGeneralStats (String geneFile, String intersectFile1, String intersectFile2, String excludeFile, String outFile) throws IOException |
|  | |
| static void | main (String[] args) throws IOException |
|  | |

## Detailed Description

Author
:   prussell

## Member Function Documentation

|  |  |  |  |
| --- | --- | --- | --- |
| static int broad.core.overlaputils.GeneSetIntersect.countOverlappers | ( | Map< String, Collection< Gene >> | *regionsToCount*, |
|  |  | Map< String, Collection< Gene >> | *regionsToOverlapWith* |
|  | ) |  |  |

static

Count number of regions that overlap at least one region in other set

Parameters
:   |  |  |
    | --- | --- |
    | regionsToCount | Regions to count |
    | regionsToOverlapWith | Potential overlappers |

Returns
:   The number of regions in the first set that overlap at least one region in other set

Here is the call graph for this function:

Here is the caller graph for this function:

|  |  |  |  |
| --- | --- | --- | --- |
| static int broad.core.overlaputils.GeneSetIntersect.countOverlappers | ( | Map< String, Collection< Gene >> | *regionsToCount*, |
|  |  | Map< String, Collection< Gene >> | *regionsToOverlapWith*, |
|  |  | Map< String, Collection< Gene >> | *excludeSet* |
|  | ) |  |  |

static

Count number of regions that overlap at least one region in other set

Parameters
:   |  |  |
    | --- | --- |
    | regionsToCount | Regions to count |
    | regionsToOverlapWith | Potential overlappers |
    | excludeSet | Exclude regions from first set if they overlap a region in this set |

Returns
:   The number of regions in the first set that overlap at least one region in other set

Here is the call graph for this function:

|  |  |  |  |
| --- | --- | --- | --- |
| static Map<String, Collection<Gene> > broad.core.overlaputils.GeneSetIntersect.getOverlap | ( | Map< String, Collection< Gene >> | *genes*, |
|  |  | Map< String, Collection< Gene >> | *intersectSet* |
|  | ) |  |  |

static

Get overlap between two gene sets

Parameters
:   |  |  |
    | --- | --- |
    | genes | Genes by chromosome |
    | intersectSet | Set to intersect with by chromosome |

Returns
:   Set of Gene objects representing the overlaps

Here is the caller graph for this function:

|  |  |  |  |
| --- | --- | --- | --- |
| static Map<String, Collection<Gene> > broad.core.overlaputils.GeneSetIntersect.getOverlap | ( | Map< String, Collection< Gene >> | *genes*, |
|  |  | Map< String, Collection< Gene >> | *intersectSet*, |
|  |  | boolean | *verbose* |
|  | ) |  |  |

static

Get overlap between two gene sets

Parameters
:   |  |  |
    | --- | --- |
    | genes | Genes by chromosome |
    | intersectSet | Set to intersect with by chromosome |
    | verbose | Print messages |

Returns
:   Set of Gene objects representing the overlaps

Here is the call graph for this function:

|  |  |  |  |
| --- | --- | --- | --- |
| static Map<String, Collection<Gene> > broad.core.overlaputils.GeneSetIntersect.getOverlap | ( | Map< String, Collection< Gene >> | *genes*, |
|  |  | Map< String, Collection< Gene >> | *intersectSet*, |
|  |  | Map< String, Collection< Gene >> | *excludeSet* |
|  | ) |  |  |

static

Get overlap between two gene sets and automatically exclude all genes in a set

Parameters
:   |  |  |
    | --- | --- |
    | genes | Genes by chromosome |
    | intersectSet | Set to intersect with by chromosome |
    | excludeSet | Exclude genes if they overlap a region from this set |

Returns
:   Set of Gene objects representing the overlaps

Here is the call graph for this function:

|  |  |  |  |
| --- | --- | --- | --- |
| static Map<String, Collection<Gene> > broad.core.overlaputils.GeneSetIntersect.getOverlap | ( | Map< String, Collection< Gene >> | *genes*, |
|  |  | Map< String, Collection< Gene >> | *intersectSet*, |
|  |  | Map< String, Collection< Gene >> | *excludeSet*, |
|  |  | boolean | *verbose* |
|  | ) |  |  |

static

Get overlap between two gene sets and automatically exclude all genes in a set

Parameters
:   |  |  |
    | --- | --- |
    | genes | Genes by chromosome |
    | intersectSet | Set to intersect with by chromosome |
    | excludeSet | Exclude genes from first gene set if they overlap a gene from this set |
    | verbose | Print messages |

Returns
:   Set of Gene objects representing the overlaps

|  |  |  |  |
| --- | --- | --- | --- |
| static Map<String, Collection<Gene> > broad.core.overlaputils.GeneSetIntersect.getOverlapWithRandomizedPositions | ( | Map< String, Collection< Gene >> | *regions*, |
|  |  | Map< String, Collection< Gene >> | *intersectGenes* |
|  | ) |  |  |

static

Intersect a set of regions with another set of genes and randomize positions of the regions within each gene

Parameters
:   |  |  |
    | --- | --- |
    | regions | The regions of interest |
    | intersectGenes | The genes to intersect with and randomize within |

Returns
:   Regions that have been intersected with the gene set and then randomized within each intersect gene

Here is the caller graph for this function:

|  |  |  |  |
| --- | --- | --- | --- |
| static Map<String, Collection<Gene> > broad.core.overlaputils.GeneSetIntersect.getOverlapWithRandomizedPositions | ( | Map< String, Collection< Gene >> | *regions*, |
|  |  | Map< String, Collection< Gene >> | *intersectGenes*, |
|  |  | Map< String, Collection< Gene >> | *excludeFeatures* |
|  | ) |  |  |

static

Intersect a set of regions with another set of genes and randomize positions of the regions within each gene

Parameters
:   |  |  |
    | --- | --- |
    | regions | The regions of interest |
    | intersectGenes | The genes to intersect with and randomize within |
    | excludeFeatures | Do not consider features that overlap one of these |

Returns
:   Regions that have been intersected with the gene set and then randomized within each intersect gene

|  |  |  |  |  |  |
| --- | --- | --- | --- | --- | --- |
| static void broad.core.overlaputils.GeneSetIntersect.main | ( | String[] | *args* | ) | throws IOException |

static

Parameters
:   |  |  |
    | --- | --- |
    | args |  |

Exceptions
:   |  |  |
    | --- | --- |
    | IOException |  |

Here is the call graph for this function:

|  |  |  |  |
| --- | --- | --- | --- |
| static Map<String, Map<Gene, Collection<Gene> > > broad.core.overlaputils.GeneSetIntersect.mapGenesToOverlappers | ( | Map< String, Collection< Gene >> | *genes*, |
|  |  | Map< String, Collection< Gene >> | *intersectSet* |
|  | ) |  |  |

static

Map each gene to the collection of overlappers in another set

Parameters
:   |  |  |
    | --- | --- |
    | genes | The genes by chromosome |
    | intersectSet | The other set by chromosome |

Returns
:   By chromosome: map of each gene that has overlappers in the other set to its overlappers

|  |  |  |  |
| --- | --- | --- | --- |
| static int broad.core.overlaputils.GeneSetIntersect.numOverlappingBases | ( | Map< String, Collection< Gene >> | *genes*, |
|  |  | Map< String, Collection< Gene >> | *intersectSet* |
|  | ) |  |  |

static

Get number of overlapping bases between two gene sets

Parameters
:   |  |  |
    | --- | --- |
    | genes | Genes by chromosome |
    | intersectSet | Set to intersect with by chromosome |

Returns
:   The total number of overlapping bases

|  |  |  |  |
| --- | --- | --- | --- |
| static int broad.core.overlaputils.GeneSetIntersect.numOverlappingBases | ( | Map< String, Collection< Gene >> | *genes*, |
|  |  | Map< String, Collection< Gene >> | *intersectSet*, |
|  |  | Map< String, Collection< Gene >> | *excludeSet* |
|  | ) |  |  |

static

Get number of overlapping bases between two gene sets and automatically exclude all genes in a set

Parameters
:   |  |  |
    | --- | --- |
    | genes | Genes by chromosome |
    | intersectSet | Set to intersect with by chromosome |
    | excludeSet | Exclude genes if they overlap a gene from this set |

Returns
:   The total number of overlapping bases

Here is the call graph for this function:

|  |  |  |  |
| --- | --- | --- | --- |
| static int broad.core.overlaputils.GeneSetIntersect.numOverlappingGenes | ( | Map< String, Collection< Gene >> | *genes*, |
|  |  | Map< String, Collection< Gene >> | *intersectSet* |
|  | ) |  |  |

static

Get number of overlapping genes between two gene sets

Parameters
:   |  |  |
    | --- | --- |
    | genes | Genes by chromosome |
    | intersectSet | Set to intersect with by chromosome |

Returns
:   The total number of overlapping bases

Here is the caller graph for this function:

|  |  |  |  |
| --- | --- | --- | --- |
| static int broad.core.overlaputils.GeneSetIntersect.numOverlappingGenes | ( | Map< String, Collection< Gene >> | *genes*, |
|  |  | Map< String, Collection< Gene >> | *intersectSet*, |
|  |  | Map< String, Collection< Gene >> | *excludeGenes* |
|  | ) |  |  |

static

Get number of overlapping genes between two gene sets and automatically exclude all genes in a set

Parameters
:   |  |  |
    | --- | --- |
    | genes | Genes by chromosome |
    | intersectSet | Set to intersect with by chromosome |
    | excludeGenes | Exclude genes from first gene set if they overlap a gene from this set |

Returns
:   The total number of overlapping bases

Here is the call graph for this function:

|  |  |  |  |
| --- | --- | --- | --- |
| static void broad.core.overlaputils.GeneSetIntersect.writeGeneralStats | ( | String | *geneFile*, |
|  |  | String | *intersectFile1*, |
|  |  | String | *intersectFile2*, |
|  |  | String | *excludeFile*, |
|  |  | String | *outFile* |
|  | ) |  | throws IOException |

static

Write basic overlap stats to a file

Parameters
:   |  |  |
    | --- | --- |
    | geneFile | Gene bed file |
    | intersectFile1 | Intersect set 1 bed file |
    | intersectFile2 | Intersect set 2 bed file |
    | excludeFile | Exclude genes if they overlap a gene in this bed file |
    | outFile | Output file of stats |

Exceptions
:   |  |  |
    | --- | --- |
    | IOException |  |

Here is the call graph for this function:

Here is the caller graph for this function:

|  |  |  |  |
| --- | --- | --- | --- |
| static void broad.core.overlaputils.GeneSetIntersect.writeOverlap | ( | String | *geneFile*, |
|  |  | String | *intersectFile*, |
|  |  | String | *outFile*, |
|  |  | boolean | *randomizePositionsWithinEachGene* |
|  | ) |  | throws IOException |

static

Get overlap between two gene sets specified in bed files and write to a file

Parameters
:   |  |  |
    | --- | --- |
    | geneFile | Bed file of genes |
    | intersectFile | Bed file of regions to intersect with |
    | outFile | Output bed file to write genes representing the overlaps |
    | randomizePositionsWithinEachGene | Whether to randomize the position of each overlap within the gene |

Exceptions
:   |  |  |
    | --- | --- |
    | IOException |  |

Here is the caller graph for this function:

|  |  |  |  |
| --- | --- | --- | --- |
| static void broad.core.overlaputils.GeneSetIntersect.writeOverlap | ( | String | *geneFile*, |
|  |  | String | *intersectFile*, |
|  |  | String | *excludeFile*, |
|  |  | String | *outFile*, |
|  |  | boolean | *randomizePositionsWithinEachGene* |
|  | ) |  | throws IOException |

static

Get overlap between two gene sets specified in bed files, automatically excluding all genes in a set, and write to a file

Parameters
:   |  |  |
    | --- | --- |
    | geneFile | Bed file of genes |
    | intersectFile | Bed file of regions to intersect with |
    | excludeFile | Exclude genes if they overlap a gene from this set |
    | outFile | Output bed file to write Genes representing the overlaps |
    | randomizePositionsWithinEachGene | Whether to randomize the position of each overlap within the gene |

Exceptions
:   |  |  |
    | --- | --- |
    | IOException |  |

Here is the call graph for this function:

|  |  |  |  |
| --- | --- | --- | --- |
| static void broad.core.overlaputils.GeneSetIntersect.writeOverlap | ( | Map< String, Collection< Gene >> | *genes*, |
|  |  | Map< String, Collection< Gene >> | *intersectSet*, |
|  |  | String | *outFile*, |
|  |  | boolean | *randomizePositionsWithinEachGene* |
|  | ) |  | throws IOException |

static

Get overlap between two gene sets and write to a file

Parameters
:   |  |  |
    | --- | --- |
    | genes | Genes by chromosome |
    | intersectSet | Set to intersect with by chromosome |
    | outFile | Output bed file to write Genes representing the overlaps |
    | randomizePositionsWithinEachGene | Whether to randomize the position of each overlap within the gene |

Exceptions
:   |  |  |
    | --- | --- |
    | IOException |  |

Here is the call graph for this function:

|  |  |  |  |
| --- | --- | --- | --- |
| static void broad.core.overlaputils.GeneSetIntersect.writeOverlap | ( | Map< String, Collection< Gene >> | *genes*, |
|  |  | Map< String, Collection< Gene >> | *intersectSet*, |
|  |  | Map< String, Collection< Gene >> | *excludeSet*, |
|  |  | String | *outFile*, |
|  |  | boolean | *randomizePositionsWithinEachGene* |
|  | ) |  | throws IOException |

static

Get overlap between two gene sets, automatically excluding all genes in a set, and write to a file

Parameters
:   |  |  |
    | --- | --- |
    | genes | Genes by chromosome |
    | intersectSet | Set to intersect with by chromosome |
    | excludeSet | Exclude genes if they overlap a gene from this set |
    | outFile | Output bed file to write Genes representing the overlaps |
    | randomizePositionsWithinEachGene | Whether to randomize the position of each overlap within the gene |

Exceptions
:   |  |  |
    | --- | --- |
    | IOException |  |

Here is the call graph for this function:

---

The documentation for this class was generated from the following file:

- java/broad/core/overlaputils/GeneSetIntersect.java


---

Generated on Fri Aug 15 2014 15:10:55 for ESAT by  

 1.8.7
